# Atezolizumab-induced neuromuscular syndrome mimicking myasthenia gravis in a patient with hepatocellular carcinoma – an area of concern

**DOI:** 10.3332/ecancer.2025.1949

**Published:** 2025-07-17

**Authors:** Mirza Rameez Samar, Mohammad Saad Salim Naviwala, Faryal Raza Abdy, Mehwish Shahzadi

**Affiliations:** 1 Department of Oncology, The Aga Khan University, Karachi 74600, Pakistan; 2 Department of Medicine, The Aga Khan University, Karachi 74600, Pakistan

**Keywords:** Atezolizumab, hepatocellular carcinoma, treatment-related adverse events, myasthenia gravis, plasma exchange, IVIG

## Abstract

**Background:**

Atezolizumab is an immune checkpoint inhibitor that has been approved for several neoplasms including advanced hepatocellular carcinoma. It is also capable of inducing adverse events involving the nervous system in susceptible individuals.

**Case presentation:**

We report a case of myasthenia gravis, occurring in a 64-year-old patient diagnosed with hepatocellular carcinoma, who within a few months of the first-line combination use of Atezolizumab and Bevacizumab, developed worsening muscular weakness. She was managed with plasma exchange and intravenous immunoglobulin with minimal response initially, but later on, deteriorated and succumbed to the condition.

**Conclusion:**

Although uncommon, the use of Atezolizumab can either give rise to or worsen a pre-existing, latent neurological condition. These neurological immune-mediated adverse events can prove to be debilitating or even life-threatening if not timely treated, thus warranting prompt investigation by the physicians for proper diagnosis and management.

## Introduction

Over the past decade, the treatment paradigm of several neoplasms has undergone metamorphosis with the inception of monoclonal antibodies, also known as immune checkpoint inhibitors (ICIs). The use of ICI in cancer patients, results in the blocking of certain molecules that lead to inactivation of T-cells with the resultant enhancement in immune response against the neoplastic cells.

Atezolizumab is a humanised immunoglobulin that works by inhibiting programmed cell death ligand-1 (PD-L1). This inhibition in turn prevents the linkage of PD-L1 expressed on the tumour cells to programmed cell death receptor-1 (PD-1) expressed on the T-cells, thus diminishing tumour-induced activation of T-cells. These ICIs in addition to inducing an immune response, are also capable of triggering treatment-related adverse events (tRAEs) in susceptible individuals by unleashing a storm of T cells [1]. These tRAEs can range from mild to life-threatening which may require treatment discontinuation with or without systemic corticosteroid administration. Not only do these vary in severity, but they could lead to a wide variety of immune-related conditions such as pneumonitis, colitis, hepatitis and auto-immune encephalitis.

Few case reports have highlighted worsening of denovo myasthenia, occurrence of secondary myasthenia, or myasthenia-like syndromes with the use of Atezolizumab [2–4]. Moreover, peripheral nervous system complications caused by Atezolizumab are also rarely reported. This report describes a case of a patient, diagnosed with hepatocellular carcinoma, who in the course of his treatment with an anti-PD-L1 antibody, was found to have myasthenia gravis (MG).

## Case presentation

**Figure 1.**
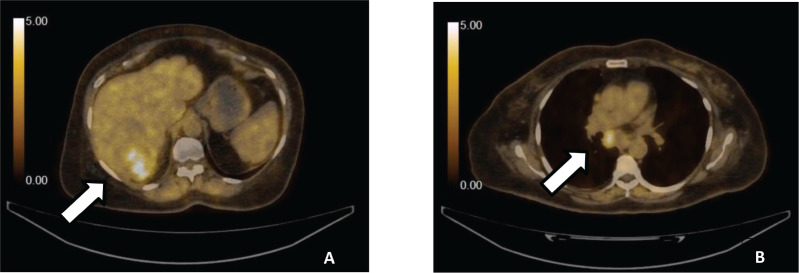
(a): PET-CT demonstrating liver with cirrhotic changes with low FDG avid necrotic hypodense lesion seen involving the right lobe. (b): Metastatic hepatocellular carcinoma showing low FDG avid subcarinal nodes.

We report a case of 64 years old patient, with an Eastern Cooperative Oncology Group 2, known to have uncontrolled type II diabetes mellitus, hypertension and hepatitis B virus-associated chronic liver disease, presented in a medical oncology clinic in July 2022 with generalised weakness and right hypochondriac pain for 4 months. Computed tomography abdomen with triphasic contrast showed a well-defined liver lesion in the right lobe with characteristic enhancement on the arterial phase followed by a washout on the delayed venous phase. Laboratory work-up revealed an elevated alpha-fetoprotein level of 4,164 IU/mL. This was followed by a staging whole-body positron emission tomography-computed tomography scan (PET-CT), which showed a hypodense lesion in the right lobe of the liver, approximately 75 × 74 × 73 mm in size, having a maximum standard uptake value (SUVmax) of 5.8 with proximal inferior vena cava thrombus along with sub-carinal, right para-cardiac and porta-hepatic lymph nodes (Figure 1).

The patient complained of right-sided shoulder pain for which a rheumatologist was consulted for the possibility of rheumatoid arthritis or any other autoimmune disease. Anti-nuclear antibodies came out to be positive whereas rheumatoid factor, extractable nuclear antigen antibodies panel and anti-double stranded DNA came out as negative. The patient was diagnosed with rotator cuff tendinitis of the right shoulder, secondary to diabetes and was cleared by her rheumatologist to proceed with systemic immunotherapy.

Belonging to Child Pugh’s Class A, systemic treatment with intravenous Atezolizumab 1,200 mg and intravenous Bevacizumab at 15 mg/kg was started, which was given every 21 days. The first cycle was given on 5th July 2022, which was well tolerated, following which 2nd cycle was given on 28th July 2022. On the 15th day of her second cycle, the patient started complaining of drooping of both eyelids which was associated with dyspnea on exertion and dysphagia for 3 days. This was followed by a visit to a neuro-physician who admitted the patient through emergency (ER) under the impression of suspected MG. In the ER, upon general physical examination, bilateral ptosis was appreciated. Upon neurological examination, the Glasgow Coma Scale was 15/15. Both the pupils were equally reactive to light. Superficial and deep reflexes were also intact. Motor examination revealed power of 3/5 in bilateral lower limbs with down-going plantar reflexes. Cough and gag reflexes were intact; however, the patient could not count up to seven in a single breath. Upon respiratory examination, decreased breath sounds were noted bilaterally. Abdominal and cardiovascular examination was unremarkable. Arterial blood gas initially showed a pH of 7.33, PCO2 of 51, PaO2 of 135, HCO3 of 25 and oxygen saturation of 98%. Non-invasive ventilatory (NIV) support was provided at 12 by 6 under Spontaneous/Timed (ST) mode, with a backup rate of 24. The medical intensive care unit team evaluated and labeled her as high risk for intubation. Forced vital capacity was measured at 4-hour intervals that were 0.38, 0.34 and 0.32 litres. An electromyogram with nerve conduction study was carried out, they did not show any decremental response or any evidence of muscle damage. Acetylcholinesterase levels were found to be <0.025 nmol/L (negative if less than 0.40 nmol/L). Anti-muscle specific kinase antibody was 0.18 U/mL (negative if less than 0.4 U/mL). The patient was treated on the lines of suspected seronegative immune-related MG and underwent 5 sessions of plasma exchange (PLEX) from 12th August 2022 to 16th August 2022. As there was not any clinical improvement, 5 doses of intravenous immunoglobulin (IVIG) were also administered from 19th August 2022 to 23rd August 2022 after which minimal clinical improvement was noticed and the patient was discharged on NIV support on the oral tablet Pyridostigmine 60 mg every 6 hourly. The patient was scheduled to follow up in neurology and oncology clinics with plan to switch to oral tyrosine kinase inhibitor by the primary oncologist upon clinical recovery; however, the patient presented in the ER with shortness of breath and worsening oxygen saturation within 48 hours. Given her debilitating condition, the family opted for comfort measures and despite being provided with supplemental oxygen support, the patient expired.

## Discussion

MG is a neuromuscular junction disorder that presents with fluctuating weakness of the skeletal muscles. Although it frequently affects ocular, facial and limb muscles, it could also involve the bulbar and/or respiratory muscles, if left untreated. The usual presentation of MG is worsening of weakness and fatigue with repetitive movement as the day progresses. It is characterised by the presence of auto-antibodies, against various receptors found on the post synaptic membrane. These are most commonly acetylcholine receptors (AChR), followed by muscle-specific kinase or less commonly, lipoprotein-related protein 4. Of these, AChR can be seen in up to 80% of the patients with immune-mediated MG [5] with average onset of MG reported within 4–6 weeks after commencing treatment[6]. A diverse group of ICIs have been approved to date. Among these Nivolumab and Pembrolizumab target PD-1, Ipilimumab targets cytotoxic T-lymphocyte-associated protein 4, whereas Durvalumab and Atezolizumab target PDL-1. Although to date, several toxicities have been reported that commonly affect bowel, liver, lung and endocrine tissue, there is also the risk of development of immunotherapy-induced neurotoxicity with use of ICIs. The neurological adverse reactions are rare in presentation, accounting for approximately 0.2%–0.4% of the cases receiving anti-PD-L1 treatment. These are mostly mild in intensity. The wide range of neurological tRAE includes several types of encephalopathies (e.g., demyelinating and vasculitic encephalopathies, posterior reversible encephalopathy syndrome), myelopathy, meningitis, peripheral neuropathy, Guillain-Barré syndrome and myasthenic syndromes [7, 8]. In a study evaluating neurotoxicity profiles of different ICIs, myositis was found to be the most frequent neuromuscular side effect [9] Being an auto-immune phenomenon, ICI can also induce myasthenia or myasthenia-like syndromes. In a review, myasthenic syndromes accounted for 14% of the neurologic tRAEs [10]. In another systematic review, the use of ICI resulted in both, flare of the primary autoimmune disease and/or new-onset tRAEs [11]. Among the ICI, anti-PD-1 was found to be more associated with MG, based on a pharmacovigilance review [12]. The management of tRAEs to ICIs is complex and requires a multidisciplinary approach. The first step, however, remains the same, that is, immediate discontinuation of the drug. Current guidelines also recommend initiation of high-dose corticosteroids (either oral or intravenous), IVIG, PLEX and immunosuppressive drugs. The use of IVIG and PLEX is usually reserved for patients who are refractory to steroids. In a single-center study evaluating 63 patients with ICI-induced MG, the use of first-line IVIG or PLEX resulted in a better response rate when compared to steroids (95% versus 63%) [6]. Although cases of new-onset MG by the use of ICIs have been brought to light, rarely do they occur secondary to use of Atezolizumab. To the best of our knowledge, this is the first reported case of Atezolizumab-induced myasthenia syndrome in our country.

## Conclusion

Immune-mediated MG is an emergent, acute illness that can be encountered in oncological practice with the use of ICI. It could prove to be lethal as a result of rapid decline in clinical presentation. Therefore, it is crucial for physicians to promptly recognise such patients, to commence therapeutic strategies timely.

## List of abbreviations

AChR, Acetylcholine; ANA, Anti-nuclear antibodies; CT, Computed tomography; ER, Emergency; ICI, Immune checkpoint inhibitors; IVIG, Intravenous immunoglobulin; MG, Myasthenia gravis; PD-1, Programmed cell death receptor-1; PD-L1, Programmed cell death ligand-1; PET-CT, Positron emission tomography-computed tomography; PLEX, Plasma exchange; SUVmax, Standard uptake value; tRAEs, Treatment-related adverse events; RA, Rheumatoid factor.

## Conflicts of interest

The authors declare that they have no competing interests.

## Funding

No specific funding has been used for manuscript writing or reporting.

## Consent for publication

Written informed consent was obtained from the patient for the publication of this case report and any accompanying images. A copy of the written consent is available for review by the Editor-in-Chief of this journal.

## Ethics approval and consent to participate

Not applicable.

## Availability of data and materials

The datasets used and/or analyzed during the current study are available from the corresponding author on reasonable request.

## Author contributions

MRS, SN and FRA gave a relevant contribution in writing the original manuscript. MRS also contributed to the conception, formatting and revision of the final manuscript. FRA, SSN and MS were involved in reviewing and providing critically important content for the manuscript. MS also reviewed the final version of the manuscript. All authors read and gave their approval for the final version to be published.
